# (2,6-Diisopropyl­phen­yl)(2-thienylmethyl­ene)amine

**DOI:** 10.1107/S1600536809006291

**Published:** 2009-02-25

**Authors:** Wolfgang Imhof

**Affiliations:** a Institute of Inorganic and Analytical Chemistry, Friedrich Schiller University, August-Bebel-Strasse 2, 07743 Jena, Germany

## Abstract

The title compound, C_17_H_21_NS, was prepared by the condensation of thio­phene-2-carbaldehyde with 2,6-diiso­propyl­aniline. It crystallizes with two mol­ecules in the asymmetric unit. The mol­ecules are inter­connected *via* a C—H⋯N hydrogen bond. The dihedral angles between the thio­phene and phenyl rings are 81.7 (7) and 85.5 (7)°.

## Related literature

For the synthetic procedure, see: Drisko & McKennis (1952 ▶); Wang *et al.* (2007 ▶). For related structures, see: Kazak *et al.* (2000 ▶); Małeki *et al.* (2007 ▶). For the organometallic chemistry of related ligands, see: Imhof (1997*a* ▶,*b* ▶).
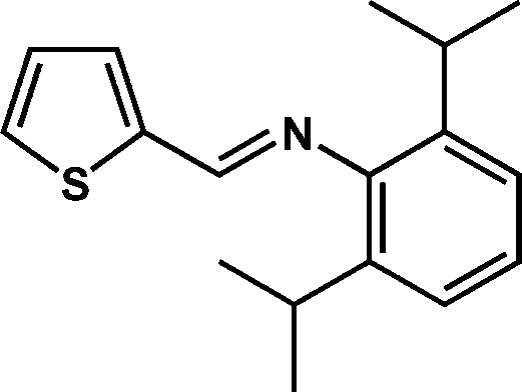


## Experimental

### 

#### Crystal data

C_17_H_21_NS
*M* *_r_* = 271.42
Monoclinic, 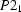

*a* = 10.0877 (9) Å
*b* = 14.275 (3) Å
*c* = 11.4503 (9) Å
β = 109.651 (8)°
*V* = 1552.8 (4) Å^3^
*Z* = 4
Mo *K*α radiation
μ = 0.20 mm^−1^
*T* = 173 K
0.51 × 0.43 × 0.33 mm

#### Data collection

Enraf–Nonius CAD-4 diffractometer
Absorption correction: ψ scan (North *et al.*, 1968 ▶) *T*_min_ = 0.884, *T*_max_ = 0.939
2222 measured reflections
2096 independent reflections
2055 reflections with *I* > 2σ(*I*)
*R*_int_ = 0.023
θ_max_ = 23.0°
3 standard reflections frequency: 120 min intensity decay: 0.02%

#### Refinement

*R*[*F* ^2^ > 2σ(*F* ^2^)] = 0.028
*wR*(*F* ^2^) = 0.079
*S* = 1.08
2096 reflections
353 parameters
1 restraint
H-atom parameters constrained
Δρ_max_ = 0.20 e Å^−3^
Δρ_min_ = −0.19 e Å^−3^

### 

Data collection: *CAD-4 EXPRESS* (Enraf–Nonius, 1994 ▶); cell refinement: *SET4* (de Boer *et al*., 1984 ▶); data reduction: *MolEN* (Enraf–Nonius, 1990 ▶); program(s) used to solve structure: *SHELXS97* (Sheldrick, 2008 ▶); program(s) used to refine structure: *SHELXL97* (Sheldrick, 2008 ▶); molecular graphics: *XP* (Siemens, 1990 ▶); software used to prepare material for publication: *SHELXL97*.

## Supplementary Material

Crystal structure: contains datablocks global, I. DOI: 10.1107/S1600536809006291/bt2882sup1.cif

Structure factors: contains datablocks I. DOI: 10.1107/S1600536809006291/bt2882Isup2.hkl

Additional supplementary materials:  crystallographic information; 3D view; checkCIF report

## Figures and Tables

**Table 1:** Hydrogen-bond geometry (Å, °)

*D*—H⋯*A*	*D*—H	H⋯*A*	*D*⋯*A*	*D*—H⋯*A*
C20—H20⋯N1	0.93	2.52	3.449 (8)	178

**Fig. 1.**
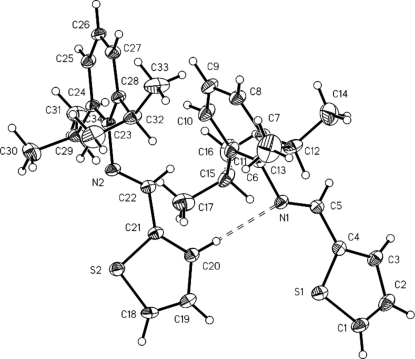
The molecular structure of the title compound, presenting the labelling scheme and 30% probability displacement ellipsoids for non-H atoms.

## supplementary crystallographic information

### Comment

In the course of a study on stoichiometric and catalytic C—H activation reactions of imines derived from 2- or 3-thiophene-carbaldehydes and aniline derivatives (Imhof, 1997*a*, 1997*b*) we synthesized the title compound that exhibits two bulky *ortho*-substituents at the phenyl ring. The compound crystallizes  with two molecules per asymmetric unit. These molecules are interconnected by hydrogen bonds between the imine nitrogen atom as the acceptor and one of the thiophene C—H functions as the hydrogen bond donor (Table 1). Bond lengths and angles correspond to values that have been reported for related imines from 2-thiophenecarbaldehyde and aniline derivatives (Kazak *et al.*, 2000; Małeki *et al.*, 2007). The dihedral angles between the thiophene and the phenyl rings measure to 98.3 (7)° and 94.5 (7)°, respectively. These values are significantly higher than those observed for compounds without bulky *ortho*-substituents (Kazak *et al.*, 2000: 20.8 (1)°; Małeki *et al.*, 2007: 49.38 (6)°).

### Experimental

The title compound was prepared in analogy to a literature method (Drisko & McKennis, 1952). The synthesis of the compound has also been recently described as an intermediate in the synthesis of the corresponding amine (Wang *et al.*, 2007). The ^1^H-NMR spectrum of the title compound is identical to the one described in the latter publication. Single crystals are produced from a solution of the compound in light petroleum (b.p. 40–60°) and dichloromethane (20:1) at -20°. MS (EI) [m/*z*, %]: 271 (*M*^+^, 76), 256 (*M*^+^ - Me, 59), 214 (C_13_H_12_NS^+^, 100), 199 (C_12_H_9_NS^+^, 17), 172 (C_10_H_6_NS^+^, 25), 146 (C_9_H_6_S^+^, 33), 132 (C_8_H_4_S^+^, 25), 115 (C_9_H_7_^+^, 15), 97 (C_5_H_5_S^+^, 36), 91 (C_7_H_7_^+^, 19), 77 (C_6_H_5_^+^, 13), 53 (C_4_H_5_^+^, 8), 41 (C_3_H_5_^+^, 14); IR (nujol mull) [cm^-1^]: 1629 (CH=N); ^13^C-NMR (CDCl_3_, 298 K) [p.p.m.]: 23.5 (CH_3_), 28.0 (CH), 123.0 (C_ar_H), 124.3 (C_ar_H), 127.7 (C_ar_H), 130.2 (C_ar_H), 131.6 (C_ar_H), 137.9 (C_ar_), 142.6 (C_ar_), 148.6 (C_ar_), 154.9 (N=CH).

### Refinement

Hydrogen atoms were positioned geometrically at distances of 0.93 Å for aromatic C—H functions and the imine C—H group, 0.98 Å for aliphatic C—H bonds and 0.96 Å for methyl groups and were refined riding on their parent atoms with isotropic displacement parameters of 1.2 times the corresponding values of their parent atoms. In the absence of significant anomalous dispersion effects, Friedel pairs were averaged.

### Crystal data

**Table:**

C_17_H_21_NS	*F*(000) = 584
*M**_r_* = 271.42	*D*_x_ = 1.161 Mg m^−^^3^
Monoclinic, *P*2_1_	Mo *K*α radiation, λ = 0.71073 Å
Hall symbol: P 2yb	Cell parameters from 25 reflections
*a* = 10.0877 (9) Å	θ = 36.5–42.8°
*b* = 14.275 (3) Å	µ = 0.20 mm^−^^1^
*c* = 11.4503 (9) Å	*T* = 173 K
β = 109.651 (8)°	Cube, yellow
*V* = 1552.8 (4) Å^3^	0.51 × 0.43 × 0.33 mm
*Z* = 4	

### Data collection

**Table:**

Enraf–Nonius CAD-4 diffractometer	2055 reflections with *I* > 2σ(*I*)
Radiation source: fine-focus sealed tube	*R*_int_ = 0.023
graphite	θ_max_ = 23.0°, θ_min_ = 1.9°
ω/2θ scans	*h* = −11→10
Absorption correction: ψ scan (North *et al.*, 1968)	*k* = 0→15
*T*_min_ = 0.884, *T*_max_ = 0.939	*l* = 0→12
2222 measured reflections	3 standard reflections every 120 min
2096 independent reflections	intensity decay: 0.02%

### Refinement

**Table:**

Refinement on *F*^2^	Secondary atom site location: difference Fourier map
Least-squares matrix: full	Hydrogen site location: inferred from neighbouring sites
*R*[*F*^2^ > 2σ(*F*^2^)] = 0.028	H-atom parameters constrained
*wR*(*F*^2^) = 0.079	*w* = 1/[σ^2^(*F*_o_^2^) + (0.0606*P*)^2^ + 0.2045*P*] where *P* = (*F*_o_^2^ + 2*F*_c_^2^)/3
*S* = 1.08	(Δ/σ)_max_ < 0.001
2096 reflections	Δρ_max_ = 0.20 e Å^−^^3^
353 parameters	Δρ_min_ = −0.19 e Å^−^^3^
1 restraint	Extinction correction: *SHELXL97* (Sheldrick, 2008), Fc^*^=kFc[1+0.001xFc^2^λ^3^/sin(2θ)]^-1/4^
Primary atom site location: structure-invariant direct methods	Extinction coefficient: 0.0222 (27)

### Special details

**Table:**

Geometry. All e.s.d.'s (except the e.s.d. in the dihedral angle between two l.s. planes) are estimated using the full covariance matrix. The cell e.s.d.'s are taken into account individually in the estimation of e.s.d.'s in distances, angles and torsion angles; correlations between e.s.d.'s in cell parameters are only used when they are defined by crystal symmetry. An approximate (isotropic) treatment of cell e.s.d.'s is used for estimating e.s.d.'s involving l.s. planes.
Refinement. Refinement on *F*^2^ for ALL reflections except for 0 with very negative *F*^2^ or flagged by the user for potential systematic errors. Weighted *R*-factors *wR* and all goodnesses of fit *S* are based on *F*^2^, conventional *R*-factors *R* are based on *F*, with *F* set to zero for negative *F*^2^. The observed criterion of *F*^2^ > σ(*F*^2^) is used only for calculating _R_factor_obs *etc*. and is not relevant to the choice of reflections for refinement. *R*-factors based on *F*^2^ are statistically about twice as large as those based on *F*, and *R*- factors based on ALL data will be even larger.

### Fractional atomic coordinates and isotropic or equivalent isotropic displacement parameters (Å^2^)

**Table:**

	*x*	*y*	*z*	*U*_iso_*/*U*_eq_	
S1	0.92670 (8)	0.27773 (6)	0.17143 (7)	0.0403 (2)	
C1	0.8043 (4)	0.3644 (2)	0.1137 (3)	0.0455 (8)	
H1	0.8191	0.4169	0.0716	0.055*	
C2	0.6842 (4)	0.3472 (2)	0.1367 (3)	0.0441 (8)	
H2	0.6065	0.3868	0.1122	0.053*	
C3	0.6886 (3)	0.2627 (2)	0.2017 (3)	0.0373 (7)	
H3	0.6143	0.2407	0.2250	0.045*	
C4	0.8135 (3)	0.2166 (2)	0.2268 (2)	0.0304 (7)	
C5	0.8510 (3)	0.1257 (2)	0.2850 (2)	0.0290 (6)	
H5	0.7887	0.0959	0.3170	0.035*	
N1	0.9657 (2)	0.08570 (16)	0.2936 (2)	0.0307 (6)	
C6	0.9982 (3)	−0.0052 (2)	0.3474 (3)	0.0303 (6)	
C7	1.0936 (3)	−0.0118 (2)	0.4707 (3)	0.0332 (7)	
C8	1.1362 (3)	−0.1004 (2)	0.5183 (3)	0.0402 (7)	
H8	1.1975	−0.1061	0.5994	0.048*	
C9	1.0900 (4)	−0.1802 (2)	0.4487 (3)	0.0459 (8)	
H9	1.1204	−0.2389	0.4822	0.055*	
C10	0.9987 (4)	−0.1719 (2)	0.3292 (3)	0.0481 (9)	
H10	0.9695	−0.2259	0.2820	0.058*	
C11	0.9485 (3)	−0.0855 (2)	0.2764 (3)	0.0371 (7)	
C12	1.1462 (3)	0.0744 (2)	0.5499 (3)	0.0425 (8)	
H12	1.1123	0.1299	0.4980	0.051*	
C13	1.3055 (5)	0.0779 (4)	0.6001 (6)	0.0936 (16)	
H13A	1.3415	0.0749	0.5326	0.112*	
H13B	1.3405	0.0258	0.6547	0.112*	
H13C	1.3353	0.1354	0.6449	0.112*	
C14	1.0880 (6)	0.0770 (3)	0.6541 (5)	0.0860 (15)	
H14A	1.1285	0.1288	0.7079	0.103*	
H14B	1.1105	0.0196	0.7002	0.103*	
H14C	0.9876	0.0844	0.6211	0.103*	
C15	0.8490 (4)	−0.0815 (2)	0.1424 (3)	0.0483 (9)	
H15	0.8103	−0.0180	0.1261	0.058*	
C16	0.7261 (4)	−0.1501 (3)	0.1206 (4)	0.0586 (10)	
H16A	0.6602	−0.1416	0.0384	0.070*	
H16B	0.6800	−0.1386	0.1802	0.070*	
H16C	0.7611	−0.2132	0.1296	0.070*	
C17	0.9284 (4)	−0.1013 (3)	0.0537 (3)	0.0595 (10)	
H17A	0.8637	−0.1017	−0.0299	0.071*	
H17B	0.9739	−0.1611	0.0728	0.071*	
H17C	0.9979	−0.0534	0.0621	0.071*	
S2	1.45567 (7)	0.01922 (5)	0.12971 (7)	0.0362 (2)	
C18	1.3736 (3)	0.1201 (2)	0.0597 (3)	0.0364 (7)	
H18	1.4044	0.1563	0.0064	0.044*	
C19	1.2598 (3)	0.1407 (2)	0.0909 (3)	0.0376 (7)	
H19	1.2027	0.1927	0.0613	0.045*	
C20	1.2366 (3)	0.0744 (2)	0.1739 (3)	0.0338 (7)	
H20	1.1629	0.0785	0.2052	0.041*	
C21	1.3337 (3)	0.0036 (2)	0.2034 (2)	0.0299 (6)	
C22	1.3372 (3)	−0.0769 (2)	0.2809 (3)	0.0306 (7)	
H22	1.2697	−0.0816	0.3194	0.037*	
N2	1.4285 (3)	−0.14133 (17)	0.2987 (2)	0.0330 (6)	
C23	1.4182 (3)	−0.2197 (2)	0.3739 (2)	0.0284 (6)	
C24	1.3357 (3)	−0.2969 (2)	0.3184 (3)	0.0298 (6)	
C25	1.3289 (3)	−0.3717 (2)	0.3932 (3)	0.0336 (7)	
H25	1.2725	−0.4229	0.3582	0.040*	
C26	1.4047 (3)	−0.3717 (2)	0.5197 (3)	0.0346 (7)	
H26	1.3980	−0.4222	0.5688	0.041*	
C27	1.4899 (3)	−0.2964 (2)	0.5719 (3)	0.0351 (7)	
H27	1.5428	−0.2977	0.6559	0.042*	
C28	1.4983 (3)	−0.2185 (2)	0.5012 (3)	0.0318 (6)	
C29	1.2653 (3)	−0.3020 (2)	0.1779 (3)	0.0349 (7)	
H29	1.2322	−0.2389	0.1487	0.042*	
C30	1.3730 (4)	−0.3295 (3)	0.1190 (3)	0.0516 (9)	
H30A	1.4096	−0.3905	0.1478	0.062*	
H30B	1.4484	−0.2847	0.1413	0.062*	
H30C	1.3294	−0.3306	0.0304	0.062*	
C31	1.1389 (3)	−0.3669 (3)	0.1358 (3)	0.0480 (8)	
H31A	1.0931	−0.3608	0.0479	0.058*	
H31B	1.0741	−0.3506	0.1777	0.058*	
H31C	1.1694	−0.4305	0.1553	0.058*	
C32	1.5953 (3)	−0.1370 (2)	0.5574 (3)	0.0417 (8)	
H32	1.5458	−0.0794	0.5207	0.050*	
C33	1.6322 (5)	−0.1290 (3)	0.6970 (3)	0.0681 (12)	
H33A	1.6913	−0.0753	0.7265	0.082*	
H33B	1.6812	−0.1844	0.7360	0.082*	
H33C	1.5475	−0.1222	0.7168	0.082*	
C34	1.7288 (4)	−0.1426 (3)	0.5227 (4)	0.0704 (12)	
H34A	1.7039	−0.1416	0.4340	0.084*	
H34B	1.7781	−0.1997	0.5546	0.084*	
H34C	1.7884	−0.0901	0.5576	0.084*	

### Atomic displacement parameters (Å^2^)

**Table:**

	*U*^11^	*U*^22^	*U*^33^	*U*^12^	*U*^13^	*U*^23^
S1	0.0442 (4)	0.0359 (4)	0.0387 (4)	0.0012 (4)	0.0110 (3)	0.0054 (4)
C1	0.065 (2)	0.0341 (18)	0.0314 (17)	0.0049 (17)	0.0084 (16)	0.0048 (15)
C2	0.0512 (19)	0.0363 (18)	0.0319 (17)	0.0130 (15)	−0.0027 (15)	0.0002 (15)
C3	0.0383 (15)	0.0336 (18)	0.0328 (16)	0.0051 (14)	0.0023 (13)	−0.0005 (14)
C4	0.0334 (14)	0.0325 (16)	0.0187 (14)	0.0017 (12)	0.0000 (12)	−0.0056 (13)
C5	0.0338 (14)	0.0297 (15)	0.0189 (14)	−0.0001 (13)	0.0028 (11)	−0.0028 (13)
N1	0.0334 (13)	0.0268 (13)	0.0280 (13)	0.0034 (11)	0.0051 (10)	0.0002 (11)
C6	0.0343 (14)	0.0281 (16)	0.0286 (16)	0.0019 (12)	0.0109 (12)	0.0022 (13)
C7	0.0403 (16)	0.0293 (16)	0.0282 (16)	0.0003 (13)	0.0090 (13)	0.0033 (13)
C8	0.0479 (17)	0.0363 (17)	0.0280 (16)	0.0050 (15)	0.0018 (14)	0.0030 (15)
C9	0.064 (2)	0.0281 (17)	0.0399 (19)	0.0096 (16)	0.0093 (16)	0.0048 (15)
C10	0.064 (2)	0.0284 (17)	0.044 (2)	0.0051 (16)	0.0069 (17)	−0.0067 (15)
C11	0.0416 (16)	0.0295 (16)	0.0340 (17)	0.0049 (13)	0.0045 (14)	−0.0049 (14)
C12	0.0499 (18)	0.0313 (17)	0.0337 (16)	−0.0038 (15)	−0.0023 (14)	0.0059 (15)
C13	0.065 (3)	0.080 (4)	0.124 (4)	−0.029 (3)	0.016 (3)	−0.031 (3)
C14	0.140 (4)	0.055 (3)	0.081 (3)	−0.010 (3)	0.062 (3)	−0.028 (3)
C15	0.058 (2)	0.0320 (17)	0.0383 (19)	0.0107 (16)	−0.0054 (16)	−0.0065 (15)
C16	0.0452 (19)	0.062 (2)	0.056 (2)	0.0053 (17)	0.0012 (18)	−0.016 (2)
C17	0.069 (2)	0.064 (2)	0.0341 (18)	−0.008 (2)	0.0018 (16)	0.0017 (19)
S2	0.0377 (4)	0.0345 (4)	0.0361 (4)	0.0016 (3)	0.0120 (3)	0.0049 (3)
C18	0.0454 (17)	0.0320 (16)	0.0280 (15)	−0.0045 (14)	0.0073 (13)	0.0094 (14)
C19	0.0380 (16)	0.0284 (16)	0.0393 (17)	0.0013 (14)	0.0034 (14)	0.0053 (15)
C20	0.0354 (15)	0.0311 (16)	0.0320 (15)	0.0005 (13)	0.0074 (12)	0.0013 (13)
C21	0.0335 (14)	0.0277 (15)	0.0216 (14)	−0.0030 (12)	0.0003 (11)	0.0013 (13)
C22	0.0325 (15)	0.0292 (15)	0.0257 (15)	−0.0037 (13)	0.0038 (12)	0.0001 (13)
N2	0.0393 (13)	0.0289 (13)	0.0270 (13)	0.0025 (11)	0.0060 (11)	0.0055 (11)
C23	0.0325 (13)	0.0270 (14)	0.0243 (14)	0.0045 (13)	0.0076 (12)	0.0066 (13)
C24	0.0343 (14)	0.0282 (15)	0.0248 (15)	0.0058 (12)	0.0070 (12)	0.0067 (13)
C25	0.0398 (15)	0.0281 (15)	0.0296 (16)	0.0006 (14)	0.0072 (13)	−0.0006 (14)
C26	0.0493 (16)	0.0261 (15)	0.0289 (16)	0.0040 (14)	0.0138 (13)	0.0061 (13)
C27	0.0437 (16)	0.0370 (17)	0.0205 (15)	0.0036 (14)	0.0053 (12)	0.0029 (14)
C28	0.0379 (13)	0.0261 (14)	0.0290 (14)	0.0035 (14)	0.0080 (11)	0.0041 (14)
C29	0.0394 (16)	0.0342 (16)	0.0240 (16)	0.0010 (13)	0.0013 (13)	0.0053 (13)
C30	0.0513 (19)	0.076 (3)	0.0222 (16)	0.0061 (18)	0.0056 (14)	0.0009 (17)
C31	0.0544 (18)	0.051 (2)	0.0296 (16)	−0.0086 (18)	0.0019 (14)	0.0020 (17)
C32	0.0548 (19)	0.0315 (17)	0.0286 (17)	−0.0016 (15)	0.0007 (15)	0.0032 (14)
C33	0.108 (3)	0.047 (2)	0.0354 (19)	−0.028 (2)	0.005 (2)	−0.0012 (18)
C34	0.057 (2)	0.069 (3)	0.073 (3)	−0.021 (2)	0.006 (2)	−0.011 (2)

### Geometric parameters (Å, °)

**Table:**

S1—C1	1.715 (3)	S2—C18	1.719 (3)
S1—C4	1.719 (3)	S2—C21	1.724 (3)
C1—C2	1.345 (5)	C18—C19	1.345 (4)
C1—H1	0.9300	C18—H18	0.9300
C2—C3	1.411 (5)	C19—C20	1.415 (4)
C2—H2	0.9300	C19—H19	0.9300
C3—C4	1.364 (4)	C20—C21	1.368 (4)
C3—H3	0.9300	C20—H20	0.9300
C4—C5	1.449 (4)	C21—C22	1.445 (4)
C5—N1	1.264 (3)	C22—N2	1.269 (4)
C5—H5	0.9300	C22—H22	0.9300
N1—C6	1.427 (4)	N2—C23	1.437 (4)
C6—C11	1.398 (4)	C23—C24	1.398 (4)
C6—C7	1.419 (4)	C23—C28	1.409 (4)
C7—C8	1.387 (5)	C24—C25	1.385 (4)
C7—C12	1.515 (4)	C24—C29	1.526 (4)
C8—C9	1.379 (5)	C25—C26	1.393 (4)
C8—H8	0.9300	C25—H25	0.9300
C9—C10	1.374 (5)	C26—C27	1.381 (4)
C9—H9	0.9300	C26—H26	0.9300
C10—C11	1.392 (5)	C27—C28	1.395 (5)
C10—H10	0.9300	C27—H27	0.9300
C11—C15	1.527 (4)	C28—C32	1.517 (4)
C12—C14	1.498 (6)	C29—C30	1.511 (5)
C12—C13	1.514 (5)	C29—C31	1.518 (5)
C12—H12	0.9800	C29—H29	0.9800
C13—H13A	0.9600	C30—H30A	0.9600
C13—H13B	0.9600	C30—H30B	0.9600
C13—H13C	0.9600	C30—H30C	0.9600
C14—H14A	0.9600	C31—H31A	0.9600
C14—H14B	0.9600	C31—H31B	0.9600
C14—H14C	0.9600	C31—H31C	0.9600
C15—C17	1.517 (5)	C32—C33	1.519 (5)
C15—C16	1.533 (5)	C32—C34	1.530 (5)
C15—H15	0.9800	C32—H32	0.9800
C16—H16A	0.9600	C33—H33A	0.9600
C16—H16B	0.9600	C33—H33B	0.9600
C16—H16C	0.9600	C33—H33C	0.9600
C17—H17A	0.9600	C34—H34A	0.9600
C17—H17B	0.9600	C34—H34B	0.9600
C17—H17C	0.9600	C34—H34C	0.9600
			
C1—S1—C4	91.53 (16)	C18—S2—C21	91.46 (14)
C2—C1—S1	111.8 (3)	C19—C18—S2	112.3 (2)
C2—C1—H1	124.1	C19—C18—H18	123.8
S1—C1—H1	124.1	S2—C18—H18	123.8
C1—C2—C3	112.9 (3)	C18—C19—C20	112.3 (3)
C1—C2—H2	123.6	C18—C19—H19	123.8
C3—C2—H2	123.6	C20—C19—H19	123.8
C4—C3—C2	112.8 (3)	C21—C20—C19	113.2 (3)
C4—C3—H3	123.6	C21—C20—H20	123.4
C2—C3—H3	123.6	C19—C20—H20	123.4
C3—C4—C5	127.5 (3)	C20—C21—C22	127.3 (3)
C3—C4—S1	111.0 (2)	C20—C21—S2	110.7 (2)
C5—C4—S1	121.4 (2)	C22—C21—S2	121.9 (2)
N1—C5—C4	122.0 (3)	N2—C22—C21	122.8 (3)
N1—C5—H5	119.0	N2—C22—H22	118.6
C4—C5—H5	119.0	C21—C22—H22	118.6
C5—N1—C6	121.0 (3)	C22—N2—C23	117.8 (2)
C11—C6—C7	121.0 (3)	C24—C23—C28	121.8 (3)
C11—C6—N1	120.6 (2)	C24—C23—N2	119.5 (2)
C7—C6—N1	118.1 (2)	C28—C23—N2	118.6 (3)
C8—C7—C6	117.9 (3)	C25—C24—C23	118.1 (3)
C8—C7—C12	120.4 (2)	C25—C24—C29	120.8 (3)
C6—C7—C12	121.7 (3)	C23—C24—C29	120.8 (3)
C9—C8—C7	121.8 (3)	C24—C25—C26	121.3 (3)
C9—C8—H8	119.1	C24—C25—H25	119.3
C7—C8—H8	119.1	C26—C25—H25	119.3
C10—C9—C8	119.2 (3)	C27—C26—C25	119.6 (3)
C10—C9—H9	120.4	C27—C26—H26	120.2
C8—C9—H9	120.4	C25—C26—H26	120.2
C9—C10—C11	122.2 (3)	C26—C27—C28	121.3 (3)
C9—C10—H10	118.9	C26—C27—H27	119.3
C11—C10—H10	118.9	C28—C27—H27	119.3
C10—C11—C6	117.9 (3)	C27—C28—C23	117.7 (3)
C10—C11—C15	119.4 (3)	C27—C28—C32	121.5 (3)
C6—C11—C15	122.7 (3)	C23—C28—C32	120.8 (3)
C14—C12—C7	110.2 (3)	C30—C29—C31	110.7 (3)
C14—C12—C13	110.3 (4)	C30—C29—C24	109.5 (2)
C7—C12—C13	111.6 (3)	C31—C29—C24	114.1 (3)
C14—C12—H12	108.2	C30—C29—H29	107.4
C7—C12—H12	108.2	C31—C29—H29	107.4
C13—C12—H12	108.2	C24—C29—H29	107.4
C12—C13—H13A	109.5	C29—C30—H30A	109.5
C12—C13—H13B	109.5	C29—C30—H30B	109.5
H13A—C13—H13B	109.5	H30A—C30—H30B	109.5
C12—C13—H13C	109.5	C29—C30—H30C	109.5
H13A—C13—H13C	109.5	H30A—C30—H30C	109.5
H13B—C13—H13C	109.5	H30B—C30—H30C	109.5
C12—C14—H14A	109.5	C29—C31—H31A	109.5
C12—C14—H14B	109.5	C29—C31—H31B	109.5
H14A—C14—H14B	109.5	H31A—C31—H31B	109.5
C12—C14—H14C	109.5	C29—C31—H31C	109.5
H14A—C14—H14C	109.5	H31A—C31—H31C	109.5
H14B—C14—H14C	109.5	H31B—C31—H31C	109.5
C17—C15—C11	110.4 (3)	C28—C32—C33	113.5 (3)
C17—C15—C16	110.7 (3)	C28—C32—C34	110.4 (3)
C11—C15—C16	111.2 (3)	C33—C32—C34	110.6 (3)
C17—C15—H15	108.1	C28—C32—H32	107.4
C11—C15—H15	108.1	C33—C32—H32	107.4
C16—C15—H15	108.1	C34—C32—H32	107.4
C15—C16—H16A	109.5	C32—C33—H33A	109.5
C15—C16—H16B	109.5	C32—C33—H33B	109.5
H16A—C16—H16B	109.5	H33A—C33—H33B	109.5
C15—C16—H16C	109.5	C32—C33—H33C	109.5
H16A—C16—H16C	109.5	H33A—C33—H33C	109.5
H16B—C16—H16C	109.5	H33B—C33—H33C	109.5
C15—C17—H17A	109.5	C32—C34—H34A	109.5
C15—C17—H17B	109.5	C32—C34—H34B	109.5
H17A—C17—H17B	109.5	H34A—C34—H34B	109.5
C15—C17—H17C	109.5	C32—C34—H34C	109.5
H17A—C17—H17C	109.5	H34A—C34—H34C	109.5
H17B—C17—H17C	109.5	H34B—C34—H34C	109.5
			
C4—S1—C1—C2	0.4 (3)	C21—S2—C18—C19	0.1 (2)
S1—C1—C2—C3	−0.2 (4)	S2—C18—C19—C20	0.3 (3)
C1—C2—C3—C4	−0.2 (4)	C18—C19—C20—C21	−0.5 (4)
C2—C3—C4—C5	−176.0 (3)	C19—C20—C21—C22	−177.2 (3)
C2—C3—C4—S1	0.5 (3)	C19—C20—C21—S2	0.6 (3)
C1—S1—C4—C3	−0.5 (2)	C18—S2—C21—C20	−0.4 (2)
C1—S1—C4—C5	176.2 (2)	C18—S2—C21—C22	177.6 (2)
C3—C4—C5—N1	173.4 (3)	C20—C21—C22—N2	176.8 (3)
S1—C4—C5—N1	−2.8 (4)	S2—C21—C22—N2	−0.8 (4)
C4—C5—N1—C6	−177.8 (2)	C21—C22—N2—C23	−177.7 (2)
C5—N1—C6—C11	84.0 (3)	C22—N2—C23—C24	88.5 (3)
C5—N1—C6—C7	−102.3 (3)	C22—N2—C23—C28	−94.9 (3)
C11—C6—C7—C8	0.1 (4)	C28—C23—C24—C25	3.0 (4)
N1—C6—C7—C8	−173.5 (3)	N2—C23—C24—C25	179.5 (2)
C11—C6—C7—C12	−178.7 (3)	C28—C23—C24—C29	−172.0 (3)
N1—C6—C7—C12	7.6 (4)	N2—C23—C24—C29	4.5 (4)
C6—C7—C8—C9	1.0 (5)	C23—C24—C25—C26	−1.7 (4)
C12—C7—C8—C9	179.9 (3)	C29—C24—C25—C26	173.2 (3)
C7—C8—C9—C10	−0.5 (5)	C24—C25—C26—C27	−0.8 (4)
C8—C9—C10—C11	−1.4 (6)	C25—C26—C27—C28	2.2 (4)
C9—C10—C11—C6	2.5 (5)	C26—C27—C28—C23	−0.9 (4)
C9—C10—C11—C15	179.1 (3)	C26—C27—C28—C32	−178.1 (3)
C7—C6—C11—C10	−1.8 (4)	C24—C23—C28—C27	−1.7 (4)
N1—C6—C11—C10	171.7 (3)	N2—C23—C28—C27	−178.2 (3)
C7—C6—C11—C15	−178.3 (3)	C24—C23—C28—C32	175.5 (3)
N1—C6—C11—C15	−4.8 (5)	N2—C23—C28—C32	−1.0 (4)
C8—C7—C12—C14	−67.3 (4)	C25—C24—C29—C30	−97.0 (3)
C6—C7—C12—C14	111.6 (4)	C23—C24—C29—C30	77.8 (4)
C8—C7—C12—C13	55.6 (5)	C25—C24—C29—C31	27.7 (4)
C6—C7—C12—C13	−125.6 (4)	C23—C24—C29—C31	−157.5 (3)
C10—C11—C15—C17	−72.7 (4)	C27—C28—C32—C33	−23.4 (5)
C6—C11—C15—C17	103.7 (4)	C23—C28—C32—C33	159.5 (3)
C10—C11—C15—C16	50.5 (4)	C27—C28—C32—C34	101.4 (4)
C6—C11—C15—C16	−133.1 (3)	C23—C28—C32—C34	−75.7 (4)

### Hydrogen-bond geometry (Å, °)

**Table:**

*D*—H···*A*	*D*—H	H···*A*	*D*···*A*	*D*—H···*A*
C20—H20···N1	0.93	2.52	3.449 (8)	178
